# *Klebsiella pneumoniae* Phage M198 and Its Therapeutic Potential

**DOI:** 10.3390/v17010115

**Published:** 2025-01-15

**Authors:** Lika Leshkasheli, Ia Kusradze, Darejan Bolkvadze, Lia Askilashvili, Maria Chichashvili, Giorgi Tsertsvadze, Elisabed Zaldastanishvili

**Affiliations:** 1 Laboratory of Molecular Biology, G. Eliava Institute of Bacteriophages, Microbiology and Virology, 0160 Tbilisi, Georgia; l.leshkasheli@pha.ge (L.L.); d.bolkvadze@pha.ge (D.B.); laskilashvili@pha.ge (L.A.); 2 Laboratory of General Microbiology, G. Eliava Institute of Bacteriophages, Microbiology and Virology, 0160 Tbilisi, Georgia; iakusradze@pha.ge (I.K.); mchichashvili@gmail.com (M.C.); 3 Faculty of Medicine, European University, 0141 Tbilisi, Georgia; 4 Program of Ecology, Faculty of Natural Sciences and Medicine, Ilia State University, 0162 Tbilisi, Georgia; 5 Department of Immunology and Microbiology, Faculty of Exact and Natural Sciences, Ivane Javakhishvili Tbilisi State University, 0179 Tbilisi, Georgia; 6 Electron Microscopy Unit, G. Eliava Institute of Bacteriophages, Microbiology and Virology, 0160 Tbilisi, Georgia; 7 School of Science and Technology, University of Georgia, 0171 Tbilisi, Georgia

**Keywords:** *Klebsiella pneumoniae*, phage, antibiotics, synergy

## Abstract

The rapid worldwide spread of antibiotic resistance is quickly becoming an increasingly concerning problem for human healthcare. Non-antibiotic antibacterial agents are in high demand for many Gram-negative bacterial pathogens, including *Klebsiella pneumoniae*. *Klebsiella*-targeting phages are among the most promising alternative therapy options. They have already been successfully applied in a number of cases, and it is expected that the need for anti-*Klebsiella* phages will only increase in the future. This prospect highlights the need for well-characterized therapeutic phages. In this work, we describe a *K. pneumoniae* phage, which also infects strains of *Klebsiella oxytoca*. Here, we characterize phage M198 in terms of its biological and genetic properties. Since in some phage therapy cases, phages are administered in combination with antibiotics, here, we also screen for possible synergistic effects of combining phage M198 with six different antibiotics. We found that phage M198 has good lytic activity against clinical isolates; it does not have any indications of a temperate lifestyle, and it has synergistic potential when combined with some therapeutically relevant antibiotics.

## 1. Introduction

The continuous and rapid spread of antibiotic-resistant bacteria creates a worldwide healthcare crisis, which, in 2050, could result in 8.22 million deaths globally [1]. Among the bacteria that contribute significantly to AMR-related deaths and illness are those belonging to the ESKAPE group (*Enterococcus faecium*, *Staphylococcus aureus*, *Klebsiella pneumoniae*, *Acinetobacter baumannii*, *Pseudomonas aeruginosa,* and *Enterobacter* spp.) [2]. This group includes bacteria against which new antibiotics are urgently needed [3]. However, new antibiotic development has seen a drastic decline in recent decades and remains unattractive for pharmaceutical companies [4,5]. Various alternatives to antibiotics have been explored, among which phages are increasingly considered a promising option [6,7,8,9,10].

Phages have long been used in therapy against a variety of bacterial infectious diseases, including those caused by antibiotic-resistant strains [11,12]. Despite only a limited number of clinical trials addressing phage therapy, individual therapy cases are frequently reported and well documented [13,14]. These include pathogens belonging to the ESKAPE group [14,15,16,17,18].

ESKAPE group includes *Klebsiella pneumoniae*—an important Gram-negative pathogen—which causes a number of diseases in humans and remains among the difficult-to-treat pathogens due to the high prevalence of antibiotic-resistant strains [19,20]. Widespread antibiotic resistance among *K. pneumoniae* isolates has triggered an active search for alternative treatment options, including new phages. The number of reports about *Klebsiella*-targeting phages is increasing, as are phage therapy cases of both successful and unsuccessful treatments of *Klebsiella*-related infectious diseases [21,22,23,24,25,26,27,28,29,30]. Phage therapy often relies on an existing collection of therapeutic phages. Having a set of phages with complementing and overlapping host ranges covering a wide variety of clinical isolates is key to the fast selection of a suitable therapeutic phage, especially considering the diversity of medically relevant strains of *Klebsiella* [19,31]. For this pool of phages to be applied practically in therapy, they have to be well characterized. When characterizing phages for therapeutic purposes, exclusion of the carriage of virulence factor encoding genes and the absence of a lysogenic lifestyle are of utmost importance [32].

Well-characterized therapeutic phages can be used in therapy independently, in phage cocktails, or in combinations with traditional antibiotics [32,33,34,35]. Synergy, as well as antagonism between phages and antibiotics, have been reported to occur at various combinations of different pathogen-targeting phages and antibiotics in vitro [36,37,38,39,40,41]. Phage–antibiotic combinations have been explored against bacteria belonging to the ESKAPE group [34,42,43]. Clinical cases of in vivo administration of phages in combination with antibiotics are also increasingly being reported [44,45]. Despite the rising number of studies examining phage–antibiotic combinations, prediction of possible interactions remains difficult. Given the growing interest in the application of combinations, the synergistic potential of therapeutic phages could be included as an important characteristic to be assessed. Here, we, therefore, characterize *K. pneumoniae* phage M198 in terms of its biological and genetic properties as well as in terms of its potential to be combined with antibiotics.

## 2. Materials and Methods

### 2.1. Bacterial Strains and the Phage

One-hundred and one *Klebsiella pneumoniae* and two *Klebsiella oxytoca* strains were used in this study. This included two type strains from each species (*K. pneumoniae* ATCC 13883 (DSM 30104) and *K. oxytoca* ATCC 13182 (DSM 5175)), which were purchased from the German collection of microorganisms and cell cultures GmbH (DSMZ). The remaining 101 strains represent clinical isolates, which were provided by the Eliava Analytical-Diagnostic Center. Phage M198 was originally isolated from sewage water by water enrichment method [46].

### 2.2. Cultivation

The phage and all bacterial strains were routinely cultivated in liquid and solid LB medium at 37 °C without agitation. If not otherwise stated, cultures were incubated overnight (O/N). For phage propagation, bacterial suspensions from slant agar cultures were used. For the selection of susceptible target strains, a spot test was carried out on double-layer agar plates [47].

### 2.3. Electron Microscopy

Morphological assessment of intact phage particles was carried out through transmission electron microscopy (JEOL 100-SX, Jeol, Akishima-Shi, Tokyo, Japan). A total of 50 μL of a sterile-filtered phage suspension (5 × 10^11^ PFU/mL) was transferred onto carbon-coated copper grids (Electron Microscopy Sciences, Hatfield, PA, USA). Uranyl acetate was used for contrast. To visualize the morphology of phage particles in the preparation, instrumental magnification of ×40,000 was used.

### 2.4. Host Range and Efficiency of Plating

Phage host range was determined on one hundred clinical strains of *K. pneumoniae,* two type strains (*K. pneumoniae* ATCC 13883 and *K. oxytoca* ATCC 13182), and one clinical isolate of *K. oxytoca*. Lytic activity of the phage was examined by spotting 10 μL of phage suspension (5 × 10^7^ pfu/mL) on a bacterial lawn [48]. The formation of a transparent zone in the spotted area was taken as an indicator of phage multiplication on target bacterial cells. The presence of a fully transparent or semi-clear zone in the spotted area was interpreted as phage sensitivity (S), opaque or turbid lysis as intermediate susceptibility (I), and no distinguishable lysis in the spotted area was interpreted as resistance to the phage (R).

Since phage sensitivity observed by spot test could be attributed to various non-phage-multiplication factors, we also performed experiments aiming at evaluating the efficiency of plating.

The efficiency of plating was determined on 20 selected susceptible strains of *K. pneumoniae* and 2 strains of *K. oxytoca*. The titer of phage propagated on the host bacterial strain was compared to the titer of the same phage propagated on bacterial strains of interest. The double-layer agar method was used to determine the phage titer on each bacterial strain [46,47,48].

The efficiency of bacteriophage cultivation was calculated as follows:

$$E=\frac{T}{T_{0}}$$

where *E* is the efficiency, *T* is the phage titer on the bacterial strain of interest, and *T*_0_ is the phage titer on the host strain. The EOP was classified into high (≥0.5), medium (0.1–0.5), or low (0.001–0.1) [49].

### 2.5. One-Step Growth

Phage adsorption and one-step growth experiments were carried out on the original host strain, *K. pneumoniae* 198. For adsorption experiments, phage and host bacterial suspensions were mixed at a multiplicity of infection (MOI) of 0.1 and were immediately incubated at 37 °C in a water bath. For determination of the number of adsorbed phages every 5 min, 0.1 mL sample was transferred to chloroform-containing pre-cooled 9.9 mL of LB, which was immediately placed on ice for 10 min. The sample was then plated using the double-layer agar method. The percentage of adsorbed phage particles was calculated from the number of phage plaques formed, which corresponds to the number of non-adsorbed phage particles in the initial test mixture.

The time period from the moment of infection to the time-point of maximum phage adsorption (minimum number of non-adsorbed phages are experimentally recorded) represents the time required for the phage to adsorb to the host bacterium. After the determined adsorption period, the phage–bacterial mix suspension was titrated at 5–10 min intervals and plated using the double-layer agar method. The number of reproduced phages in the test suspension was determined this way. Obtained numbers indicate the parameters of a single cycle of intracellular phage development, such as the duration of the latent period and the phage burst size, which were calculated according to the literature [46,48].

### 2.6. Whole Genome Sequencing and Analysis

Phage DNA was isolated from a filtered lysate (5 × 10^11^ PFU/mL) using the Invitrogen Genomic DNA Mini Kit (Thermo Fisher Scientific, Life Technologies Corp., 5781 Van Allen Way, Carlsbad, CA, USA). Genome sequencing was carried out on the Illumina NovaSeq X platform in paired-end mode (Illumina, San Diego, CA, USA) by Macrogen Europe, based in Amsterdam, The Netherlands. A TruSeq PCR-free library preparation kit with an insert size of 350 bp was utilized. Quality control of the resulting fastq files was performed using FastQC (v0.12.1) [50], and de novo genome assembly was conducted using SPAdes v3.15.3 with default parameters [51]. Open reading frames (ORFs) were identified using GeneMark, and genome annotation was performed using Artemis [52,53]. Functional annotation was completed with PHROGs v4 [54]. A circular representation of the genome was generated using Geneious, while tRNA genes were identified with tRNAscan-SE v1.3.1 [55,56].

The following methods were used for phage genome comparison. The proteomic dendrogram was created using VIPtree [57]. Average nucleotide identity (ANI) was calculated by using the ANI calculator (OrthoANIu algorithm) [58].

The complete genome sequence has been deposited in GenBank under the accession number PQ182780.1.

### 2.7. Checkerboard Assay

Potential synergistic interactions between phage M198 and selected antibiotics were determined through a checkerboard assay. Polystyrene flat-bottomed 96-well plates were used. In each well, bacterial culture was inoculated to the starting titer of 5 × 10^5^ CFU/mL in a total volume of 150 μL. Decreasing concentrations (two-fold serial dilutions) of antibiotics were applied in rows (from 1 to 10); phages were introduced in 10-fold dilutions in columns (A to H). On each plate, one row contained bacteria with only antibiotics, and one column contained bacteria with only phages (for an exemplary plate setup, see Figure S1). Wells in-between contained phage–antibiotic combinations. Phages were always applied at seven different titers from 2 × 10^8^ PFU/mL to 2 × 10^2^ PFU/mL in 10-fold serial dilutions. This corresponds to an MOI of 0.0004 to 400.

Plates were covered with breathable film (Breathe-Easy^®^, Diversified Biotech, Inc., Dedham, MA, USA) and were incubated for 18 h at 37 °C (with 1 min long shaking every 10 min) in a microplate reader (Thermo Fisher Scientific Multiskan SkyHigh Microplate Spectrophotometer, Carlsbad, CA, USA). Optical density (OD) measurements were taken every 10 min at 600 nm.

Minimal inhibitory concentrations (MICs) of tested antibiotics were determined according to the established liquid broth microdilution protocol with modifications [59]. The phage MIC was defined similarly by determining the optimal MOI, at which bacterial growth inhibition could be determined by visual inspection (corresponds to approximately a minimum of 60% growth inhibition compared to the control).

Synergistic interactions between phages and antibiotics were evaluated by calculating fractional inhibitory concentration index (*FIC*i) values according to the following formula:

$$FICi={FIC}_{A}+{FIC}_{B}=\frac{{MIC}_{AB}}{{MIC}_{A}}+\frac{{MIC}_{BA}}{{MIC}_{B}}$$

where *MIC*_A_ and *MIC*_B_ are individual *MIC*s of antibiotics and the phage, respectively, and *MIC*_AB_ and *MIC*_BA_ are the *MIC*s of antibiotics and the phage in combinations. Interactions were defined as follows: *FIC*i ≤ 0.5 = synergy; 0.5 < *FIC*i ≤ 0.625 = potentiation; 0.625 < *FIC*i ≤ 1.0 = additivity; 1.0 < *FIC*i ≤ 4.0 = indifference; *FIC*i > 4.0 = antagonism [60,61].

Antibiotics used in this work are listed in Table 1.

## 3. Results

### 3.1. Phage M198 Morphology

**Figure 1:**
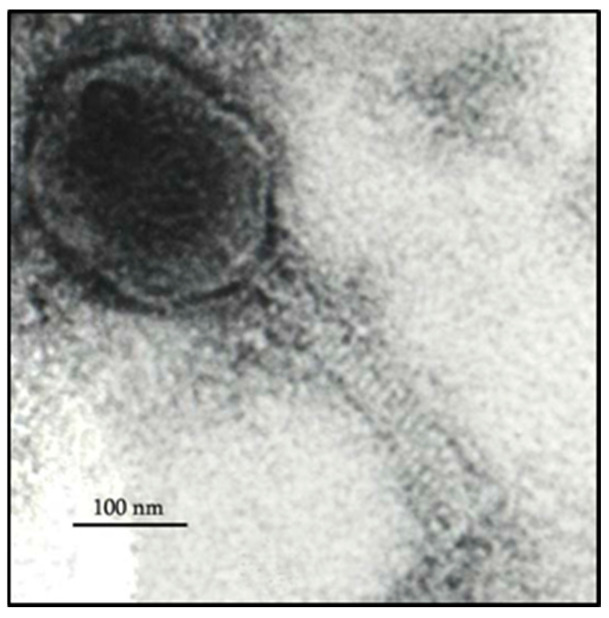
TEM image of phage M198. Scale bar corresponds to 100 nm.

According to TEM, phage M198 appears to belong to the Myoviridae morphological group. It has an icosahedral head with an approximate size of 93 × 71 nm and a contractile tail with an approximate size of 110 × 17 nm (Figure 1). These observations correspond to the findings described in Section 3.4.

### 3.2. Phage Host Range and EOP

**Figure 2:**
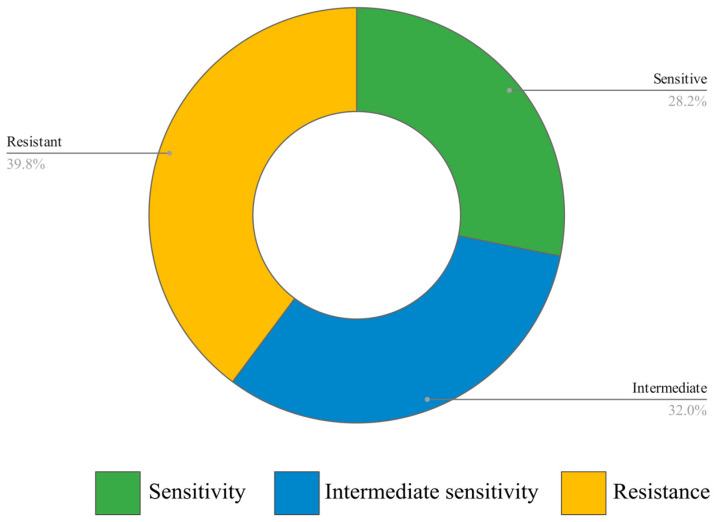
Lytic activity of phage M198 against 103 *Klebsiella* strains (101 *K. pneumoniae* strains and 2 *K. oxytoca* strains). Percentages of sensitive and intermediately strains are presented in green and blue, respectively; percentage of resistant strains is presented in yellow. Please see Table S1 for the list of tested strains.

The phage host range was initially determined on 101 *K. pneumonia* strains. Later, two *K. oxytoca* strains were also included in the study. From 103 tested stains, phage M198 was active against 62, of which 29 were fully sensitive strains, and 33 showed intermediate sensitivity (Figure 2). Phage M198 showed no lytic activity against 41 tested strains.

The efficiency of plating was calculated relative to the host strain, *K. pneumoniae* 198, where the EOP is assumed to correspond to 1. From susceptible strains, only fully sensitive ones were selected for the determination of the EOP. The phage EOP was high on 20 strains and medium on two strains (Table 2). Seven strains with EOP values of 0 are not shown.

### 3.3. Phage One-Step Growth

One-step growth parameters were determined on the original host strain of the phage—*K. pneumoniae* 198. Phage M198 adsorbs to host cells in 10 min, at which time 91% of phage particles are adsorbed. The latent period lasts for 20 min, and the lysis time is 100 min (Figure S3). Phage burst size was calculated to be 100–115 viral particles per infected cell.

### 3.4. Phage Genome Analysis

**Figure 3:**
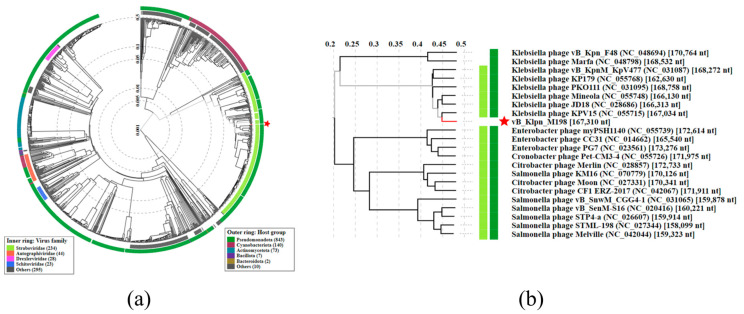
Proteomic dendrogram of phage M198. Relatedness to phages with publicly available phage genomes (**a**). A set of phages closely related to phage M198 (**b**). Proteomic dendrogram was generated by VipTree software version 4.0 [57]. Red stars indicate the location of phage M198 on the dendrogram.

According to genome analysis of phage M198 (GenBank: PQ182780.1), this phage contains a 167,310 bp long genome with 39.56% GC content. The analysis has revealed that this phage belongs to the Caudoviricetes class of the family of Staboviridae. More specifically, the Jiaodavirus genus of the Tevenvirinae subfamily. A comparison of the M198 phage genome to other known phages from public databases revealed its closeness to not just several *Klebsiella* phages but its close association with *Enterobacter* and *Salmonella* phages (Figure 3). ANI analysis revealed that the ANI between phage M198 and phage KPV15 (most similar phage from public database, GenBank accession: NC_055715.1) is 96.65%. This result indicates that both phages belong to the same species and, consequently, to the same subfamily [62].

**Figure 4:**
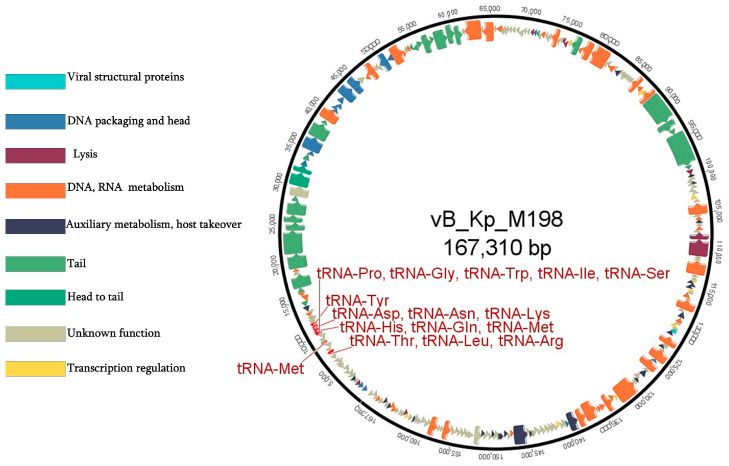
Genome map of phage M198. Colors indicate genes of different functional modules. Genes of unknown function are shown in khaki. tRNA-encoding genes are indicated. More detailed mapping of genes of known function is presented in Figure S2.

The coding percentage of the phage M198 genome corresponds to 93.6%, including introns. A total of 271 open reading frames (ORFs) were identified, of which 134 encode hypothetical proteins with unknown functions. The remaining 137 ORFs were classified into functional categories as follows: 46 ORFs involved in DNA replication, repair, and metabolism; 47 ORFs related to DNA packaging and structural assembly; 8 ORFs associated with host lysis; 25 ORFs linked to auxiliary metabolism and host takeover functions; 10 ORFs involved in transcriptional regulation; and 1 ORF encoding a membrane protein. Additionally, the genome encodes 16 tRNAs (Figure 4).

Genes associated with structural assembly, crucial for the formation of the phage head, tail, and baseplate, were identified as head proteins: major head proteins (klpn_198-047, klpn_198-056, klpn_198-057), head scaffolding proteins (klpn_198-052, klpn_198-053, klpn_198-055) and head maturation protease (klpn_198-054, klpn_198-202). Tail proteins were identified as follows: tail sheath proteins (klpn_198-046, klpn_198-049), tail tube proteins (klpn_198-050, klpn_198-077), and tail fiber proteins (klpn_198-096, klpn_198-129, klpn_198-132). Baseplate Proteins: baseplate hub subunit (klpn_198-0742), baseplate wedge subunits (klpn_198-032, klpn_198-037, klpn_198-041) and tail lysozyme (klpn_198-029).

Key DNA/RNA and metabolism-associated proteins include the following: helicases: DNA helicases (klpn_198-051, klpn_198-066, klpn_198-067); ligases: ATP-dependent DNA ligase (klpn_198-081) and RNA ligases (klpn_198-026, klpn_198-060, klpn_198-108); polymerases and primases: DNA polymerases (klpn_198-192, klpn_198-196), DNA primases (klpn_198-032, klpn_198-179), and thymidylate synthases (klpn_198-114, klpn_198-184, klpn_198-189).

Phage M198 encodes several transcriptional regulators and modulators of gene expression, among which are transcription factors: late sigma transcription factor (klpn_198-206), translation repressor (klpn_198-193), transcriptional regulators (klpn_198-126, klpn_198-161, klpn_198-165); and two anti-sigma factors were detected (klpn_198-135, klpn_198-171).

The lysis module comprises eight genes, including five associated with host cell lysis: two spanins, an endolysin, and a holin. Additionally, four genes encode lysis inhibitor proteins. Twenty-five ORFs encode auxiliary enzymes and proteins contributing to metabolic processes and host takeover.

It is important to note that no genes known to be associated with temperate phage behavior were detected, suggesting that phage M198 follows a strictly lytic life cycle.

### 3.5. Phage–Antibiotic Synergy

Four bacterial strains were selected for phage–antibiotic synergy screening: two *K. pneumoniae* strains included the original host strain of phage M198, *K. pneumoniae* 198, and a reference strain, *K. pneumoniae* ATCC 13883, as well as two *K. oxytoca* strains, one clinical isolate, *K. oxytoca* 121a, and one reference strain, *K. oxytoca* ATCC 13182. In all four cases, phages were always applied at seven different MOIs from 0.0004 to 400 in 10-fold serial dilutions. The lowest MOI corresponds to 30 phage particles per well. Even at such a low MOI, we saw total inhibition of bacterial growth for two out of four tested strains: *K. pneumoniae* 198 and *K. oxytoca* ATCC 13182. While this result highlights the efficiency of this phage, it leaves no possibility of screening for synergy in this experimental setup. We therefore concentrated on two remaining strains, *K. pneumoniae* ATCC 13883 and *K. oxytoca* 121a, since for these two strains, it was possible to determine the optimal MOI. For the purposes of synergy determination, we refer to this optimal MOI as phage MIC (Table 3).

**Figure 5:**
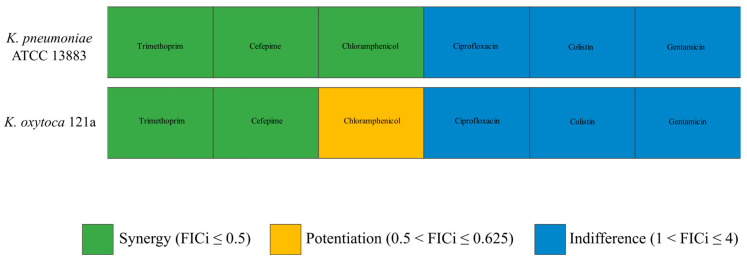
Phage–antibiotic interactions on strains *K. pneumoniae* ATCC 13883 (**top**) and *K. oxytoca* 121a (**bottom**). Synergistic relationships between phage M198 and antibiotics are shown in green; potentiation between phage M198 and chloramphenicol is presented in yellow, and indifference between phage M198 and antibiotics is shown in blue. *FIC*i values for each combination are presented in Table S2.

According to EUCAST, MICs of gentamicin-susceptible *Klebsiella* strains should not exceed 2 μg/mL [63]. In our case, both tested strains were found to be gentamicin-resistant (Table 3). Phage introduction did not lower the gentamicin MIC for either of the two strains. We, therefore, see no practical benefit in investigating the potential outcomes of such combinations. Despite being sensitive to ciprofloxacin and colistin, combining these two antibiotics with phage M198 also did not improve the antimicrobial effect of either ciprofloxacin or colistin or phage M198 (Figure 5).

Interestingly, synergistic interactions were observed between phage M198 and antibiotics with cardinally different modes of action. These included trimethoprim and cefepime for both tested strains and chloramphenicol in the case of *K. pneumoniae* ATCC 13883 (Figure 5). Interaction between phage M198 and chloramphenicol corresponds to potentiation in the case of *K. oxytoca* 121a.

Neither additivity nor antagonism were observed in any of the tested combinations.

## 4. Discussion

Here, we characterize phage M198, a phage originally isolated against *K. pneumoniae* from an environmental water sample. We examined its biological and genetic properties in order to establish its therapeutic potential.

An important characteristic of therapeutic phages is their host range. One of the very first steps in the process of therapeutic phage preparation is phage screening against the bacterial strain of interest, followed by the selection of phages to which the strain showed susceptibility [64]. From our experience, while the therapeutic application of phages with a very narrow host range is not very rare, phages with broad lytic activity will always be selected more frequently and, therefore, represent valuable members of every phage collection. In the case of phage M198, we saw that from tested strains, less than 40% were fully resistant to the phage. Despite the fact that the efficiency of plating of this phage was high on 19.4% of examined strains, intermediate sensitivity, which was observed on 32% of target strains if needed, offers the possibility of phage adaptation.

As it is known that some *K. pneumoniae* phages can infect bacterial strains of different species and even from different genera [26,30], it was unsurprising to see that the lytic activity of phage M198 covers at least two species of this genus. Considering the fact that *K. oxytoca* is rapidly emerging as an antibiotic-resistant pathogen, the need for non-antibiotic agents that target this bacterium is also expected to multiply in the future [65,66,67]. Phages remain a promising alternative to antibiotics here, but it is worth noting that besides the isolation of *K. oxytoca*-specific phages, *K. pneumoniae* phages with broad lytic activity should also be considered, especially in the processes of cocktail formulation. We, therefore, think that *K. pneumoniae* phages from existing collections should also be screened for their activity against strains of different species.

For the further establishment of an actual breadth of the lytic spectrum of phage M198, it would be beneficial to study its activity on more *K. oxytoca* strains as well as on strains from related genera, such as *Raoultella*.

Compared to using mono-phages, phage cocktails are often considered more effective, specifically because of the reduced emergence of phage resistance in targeted bacteria [68,69,70,71]. While genetic relatedness and capsular characteristics of target strains examined here were not investigated in this work, we believe that the reported lytic range suggests that phage M198 could be a good candidate for phage cocktail development. At the same time, we saw how this phage could totally inhibit the bacterial growth of selected strains without observable regrowth after 18 h. This observation puts phage M198 among those phages that can be used independently in personalized medicine.

The genomic analysis of phage M198 provides valuable insights into its genetic architecture and biological functionality. With a genome length of 167,310 bp and a GC content of 39.56%, M198 exhibits a high coding density of 93.6%, which is consistent with the compact genomes typically observed in bacteriophages. The identification of 271 open reading frames (ORFs), of which nearly 50% encode hypothetical proteins, highlights the need for further functional studies to elucidate the roles of these uncharacterized genes.

The functional classification of the remaining ORFs into key biological categories underscores the phage’s sophisticated genomic organization. Genes involved in DNA replication, repair, and metabolism, as well as those linked to DNA packaging and structural assembly, are crucial for the successful propagation of the phage within its host. The presence of 47 ORFs associated with structural proteins confirms the complex machinery required for head, tail, and baseplate assembly. Specifically, the identification of major head proteins (e.g., klpn_198-047), tail sheath proteins (e.g., klpn_198-046), and baseplate components (e.g., klpn_198-032) suggests the modular assembly typical of bacteriophages with contractile tails—in line with morphology observed by TEM [72].

Transcriptional regulation appears to be finely controlled in phage M198, with several ORFs encoding transcription factors, anti-sigma factors, and repressors. These genes likely coordinate the temporal expression of phage proteins, ensuring efficient utilization of host resources. For instance, the late sigma transcription factor (klpn_198-206) likely regulates late-stage structural and assembly gene expression.

The lysis module, consisting of holins, spanins, and endolysins, demonstrates the phage’s capacity for efficient host cell lysis. Notably, the presence of lysis inhibitor proteins suggests the potential for controlled lysis, which could enhance phage fitness by allowing optimal viral particle assembly before host cell rupture.

The genome of phage M198 encodes 16 tRNAs, which may contribute to overcoming host codon usage biases and facilitating efficient translation of phage proteins. This feature is particularly advantageous for phages infecting diverse bacterial hosts.

A significant finding is the absence of genes associated with lysogeny, indicating that phage M198 exclusively follows a lytic life cycle. This characteristic, combined with its auxiliary metabolism and host takeover genes, underscores its adaptability and potential as a therapeutic agent in phage therapy.

Overall, the genomic features of phage M198 reflect an efficient lytic lifestyle. Future research should aim to functionally characterize hypothetical proteins and explore their possible role in defining the phage interactions with host cells. This would allow us to better understand phage’s therapeutic potential and determinants of its traits, including its potential to be successfully employed in combination with traditional antibiotics.

Here, we combined phage M198 with 6 different antibiotics and evaluated their effect on two strains representing two species of *Klebsiella* genus. Cefepime and trimethoprim, which target cell wall and folate synthesis, respectively, both showed synergistic effects in combination with M198. We also examined chloramphenicol and gentamicin, both protein synthesis-targeting antibiotics but with different ribosomal targets. Interestingly, we did not see any effect of combining phage with gentamicin, while with chloramphenicol, we saw synergy or potentiation. It must be noted that all positive effects were observed at antibiotic concentrations lower than MIC. This is in line with reports reviewed previously [73,74]. If we consider phages as agents with the potential to reduce antibiotic MICs, we could hope for the reduction of side effects in those cases where synergistic combinations of phages and antibiotics are applied in therapy.

Though in this work we have seen similar effects of selected phage-antibiotic combinations on two strains belonging to different species, this observation cannot be taken as an indication that a similar picture can be expected if another phage is combined with the same antibiotics or if the same combinations are employed against other strains. We believe that such screenings are an important step to be taken before practical applications, but at the same time, further research is required to understand the mechanisms of synergy for each confirmed synergistic combination. It must be noted that we screened phage–antibiotic combinations exclusively in LB. LB does not reflect in vivo conditions, in which the same antibacterial agents would be applied in case of therapy. Therefore, even if mechanisms of synergistic interactions are known, it is important to evaluate the possible effects of environmental factors on observed interactions, especially since it is known that host factors can influence the efficacy of phage therapy [75].

Recently, phage–antibiotic synergy was observed in vivo against *Acinetobacter baumannii* [76]. This is an important step towards reconsideration of antibiotic use in cases when they would not be chosen as sole treatment agents. In our work, we see that synergy can be achieved at varying doses of antibiotics. In addition to the correct selection of combinations, dose appropriation also needs to be taken into consideration. We think that routine in vitro screening of therapeutic phages and relevant antibiotics should precede the decision to combine these two agents in patients, especially since antagonistic interactions cannot be immediately excluded [74,77].

## Figures and Tables

**Table 1:** List of commercial antibiotics used in combination with phage M198. Mode of action and highest applied concentration are indicated for each antibiotic.

Antibiotic	Mode of Action (Target)	Highest Concentration Used
Cefepime	Cell wall synthesis inhibition (penicillin-binding proteins)	1 μg/mL
Chloramphenicol	Protein synthesis inhibition (50 S ribosomal subunit)	32 μg/mL
Ciprofloxacin	DNA synthesis inhibition (gyrase & Topoisomerase IV)	1 μg/mL
Colistin	Cell wall disruption (cell membrane)	16 μg/mL
Gentamicin	Protein synthesis inhibition (30 S ribosomal subunit)	16 μg/mL
Trimethoprim	Folate synthesis inhibition (dihydrofolate reductase)	16 μg/mL

**Table 2:** Phage EOP on sensitive strains. EOP value is indicated next to each tested strain. EOP of 0.5 or higher corresponds to high efficiency, EOP value between 0.1 and 0.5 corresponds to medium efficiency, and values lower than 0.1 indicate low efficiency of plating.

Strain	EOP Value
original host	1
*K. pneumoniae* 5	1
*K. pneumoniae* 19	4
*K. pneumoniae* 27	0.2
*K. pneumoniae* 33	1
*K. pneumoniae* 41	4
*K. pneumoniae* 45	1
*K. pneumoniae* 52	0.2
*K. pneumoniae* 53	1
*K. pneumoniae* 64	2
*K. pneumoniae* 67	3
*K. pneumoniae* 68	3
*K. pneumoniae* 69	2
*K. pneumoniae* 84	3
*K. pneumoniae* 85	1
*K. pneumoniae* 90	1
*K. pneumoniae* 91	2
*K. pneumoniae* 92	1
*K. pneumoniae* 93	3
*K. pneumoniae* 94	0.5
*K. pneumoniae* ATCC 13883	0.5
*K. oxytoca* ATCC 13182	1
*K. oxytoca* 121a	0.5

**Table 3:** Minimal inhibitory concentrations (MICs) of phage M198 and of screened antibiotics on strains *K. pneumoniae* ATCC 13883 and *K. oxytoca* 121a. For antibiotics, MIC is presented in μg/mL values, while for phage M198, it represents a multiplicity of infection (MOI).

Antibacterial Agent	*K. pneumoniae* ATCC 13883	*K. oxytoca* 121a
Cefepime	0.125 μg/mL	0.0625 μg/mL
Chloramphenicol	2 μg/mL	1–2 μg/mL
Ciprofloxacin	0.0625 μg/mL	0.03125 μg/mL
Colistin	0.25 μg/mL	0.25 μg/mL
Gentamicin	4 μg/mL	4 μg/mL
Trimethoprim	1 μg/mL	1 μg/mL
Phage M198	0.04	0.004–0.04

## Data Availability

The data presented in this study are available in the article and in Supplementary Material.

## Supplementary Materials

The following supporting information can be downloaded at https://www.mdpi.com/article/10.3390/v17010115/s1. Figure S1: An example of a 96-well plate setup. Figure S2: Detailed genome map of phage M198; Figure S3: One-step growth of phage M198. Table S1: Results of phage susceptibility testing and calculated EOP values. Table S2: FICi values calculated for each phage-antibiotic combination.
