# Supplementary material for: Klebsiella pneumoniae Phage M198 and Its Therapeutic Potential
Source: Viruses. 2025 Jan 15;17(1):115. doi: 10.3390/v17010115 (PMC11768853; doi:10.3390/v17010115)
Supplement: Supplementary file 1 [file viruses-17-00115-s001.zip › viruses-3428572-supplementary.pdf]

Klebsiella pneumoniae Phage M198 and Its Therapeutic Potential
Supplementary material

Table S1. Results of phage susceptibility testing and calculated EOP values. For strains which showed intermediate sensitivity or resistance to phage M198, no EOP values are presented. Presence of a fully transparent (CL) or semi-clear zone (SCL) in the spotted area was interpreted as phage sensitivity (S); opaque or turbid lysis (OL) - as intermediate susceptibility (I); no distinguishable lysis was interpreted as resistance to the phage (R). Strains N 1-100 are clinical isolates provided by Eliava diagnostic center. They have been isolated from various sites including sputum, urine and stool.

strain #	spot test	EOP
1	SCL	0
2	SCL	0
3	R	-
4	OL	-
5	SCL	1
6	OL	-
7	OL+	-
8	R	-
9	OL	-
10	R	-
11	OL	-
12	R	-
13	OL	-
14	SCL	0
15	+	0
16	OL	-
17	R	-
18	SCL	0
19	CL	4
20	R	-
21	OL	-
22	OL	-
23	R	-
24	R	-
25	SCL	0
26	SCL	0
27	SCL	0.2
28	R	-
29	OL	-
30	OL	-
31	R	-
32	OL+	-
33	SCL	1
34	R	-
35	R	-

36	R	-
37	SCL	0
38	R	-
39	R	-
40	R	-
41	SCL	4
42	OL	-
43	OL	-
44	R	-
45	CL	1
46	OL	-
47	R	-
48	OL	-
49	R	-
50	OL	-
51	OL+	-
52	SCL	0.2
53	CL	1
54	R	-
55	R	-
56	OL	-
57	OL	-
58	OL	-
59	R	-
60	R	-
61	R	-
62	OL	-
63	OL	-
64	SCL	2
65	R	-
66	R	-
67	SCL-	3
68	SCL	3
69	SCL	2
70	R	-
71	R	-
72	OL	-
73	OL	-
74	OL	-
75	R	-
76	R	-
77	OL+	-
78	R	-
79	R	-
80	R	-
81	R	-

82	OL	-
83	R	-
84	CL	3
85	CL	1
86	OL+	-
87	R	-
88	R	-
89	R	-
90	CL	1
91	SCL+	2
92	CL	0.1
93	SCL	3
94	SCL	0.5
95	OL	-
96	OL	-
97	OL	-
98	R	-
99	R	-
100	R	-
K. pneumoniae ATCC 13883	CL	0.5
K. oxytoca ATCC 13182	SCL	1
K. oxytoca 121a	CL	0.5
104	HOST	1
E. coli ATCC 25922	R	-

Table S2. FICi values calculated for each phage-antibiotic combination, where bacterial growth inhibition was observed. Antibiotic concentration is indicated next to each antibiotic. Phage MOI always corresponds to 0.004 or 0.04. FICi ⩽0.5 = synergy; 0.5 < FICi ⩽0.625 = potentiation; 0.625 < FICi ⩽1.0 = additivity; 1.0 < FICi ⩽4.0 = indifference; FICi >4.0 = antagonism.

Antibiotic	*K. pneumoniae* ATCC 13883	*K. oxytoca* 121a
Cefepime: 0.0156 μg/ml	0.225	0.35
Cefepime: 0.0078 μg/ml	0.1625	0.225
Cefepime: 0.0039 μg/ml	0.13125	0.1625
Chloramphenicol: 0.5 μg/ml	0.35	0.35
Chloramphenicol: 0.25 μg/ml	0.225	-
Chloramphenicol: 0.125 μg/ml	0.1625	-
Trimethoprim: 0.25 μg/ml	0.35, 0.26	0.35
Trimethoprim: 0.125 μg/ml	0.225, 0.135	0.225
Trimethoprim: 0.0625 μg/ml	0.1625	0.1625
Ciprofloxacin: 0.015625 μg/ml	1.01	1.1
Gentamicin: 1 μg/ml	1.25	1.25
Colistin: 2 μg/ml	-	2
Colistin: 0.0625 μg/ml	1.015625	-

	1	2	3	4	5	6	7	8	9	10	11	12
A	Combination	Combination	Combination	Combination	Combination	Combination	Combination	Combination	Combination	2.00E+08	Blank	Culture control
B	Combination	Combination	Combination	Combination	Combination	Combination	Combination	Combination	Combination	2.00E+07	Blank	Culture control
C	Combination	Combination	Combination	Combination	Combination	Combination	Combination	Combination	Combination	2.00E+06	Blank	Culture control
D	Combination	Combination	Combination	Combination	Combination	Combination	Combination	Combination	Combination	2.00E+05	Blank	Culture control
E	Combination	Combination	Combination	Combination	Combination	Combination	Combination	Combination	Combination	2.00E+04	Blank	Culture control
F	Combination	Combination	Combination	Combination	Combination	Combination	Combination	Combination	Combination	2.00E+03	Blank	Culture control
G	Combination	Combination	Combination	Combination	Combination	Combination	Combination	Combination	Combination	2.00E+02	Blank	Culture control
H	16	8	4	2	1	0.5	0.25	0.125	0.0625	Culture control	Blank	Culture control

Figure S1. An example of a 96-well plate setup. Phages are introduced in Column 10 (A to G, shown in yellow). Phage titer is indicated. Antibiotics (For example, gentamicin) are introduced in row H (1 to 9, shown in blue). Column 11: culture medium only (shown in gray); column 12: bacteria only (shown in red). Bacteria were intoduced in all wells except for column 11 and at 5x10^5 CFU/ml. Phage titer is the same in all wells of each row (except for 11 and 12) and antibiotics concentrations are the same in all columns (except for 10, 11 and 12).

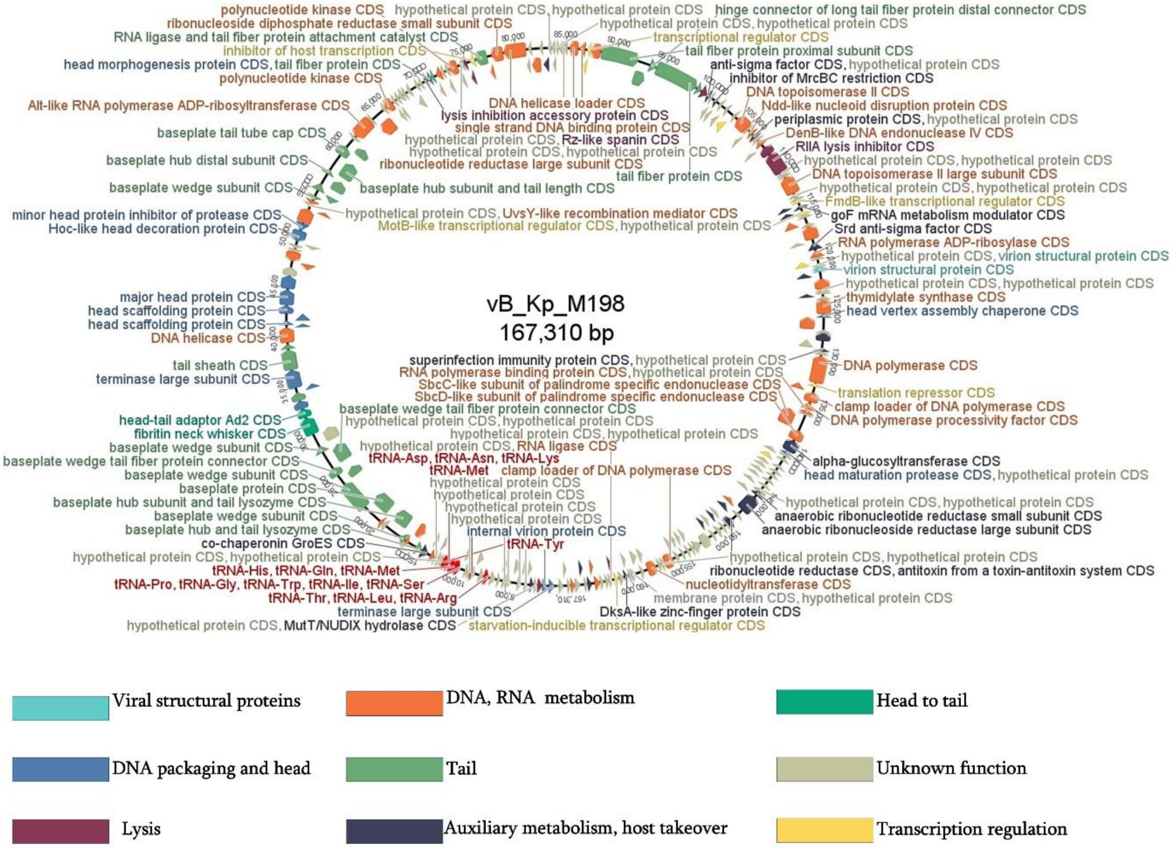

Figure S2. Detailed genome map of phage M198. Colors indicate genes of different functional modules. Genes of unknown function are shown in khaki.

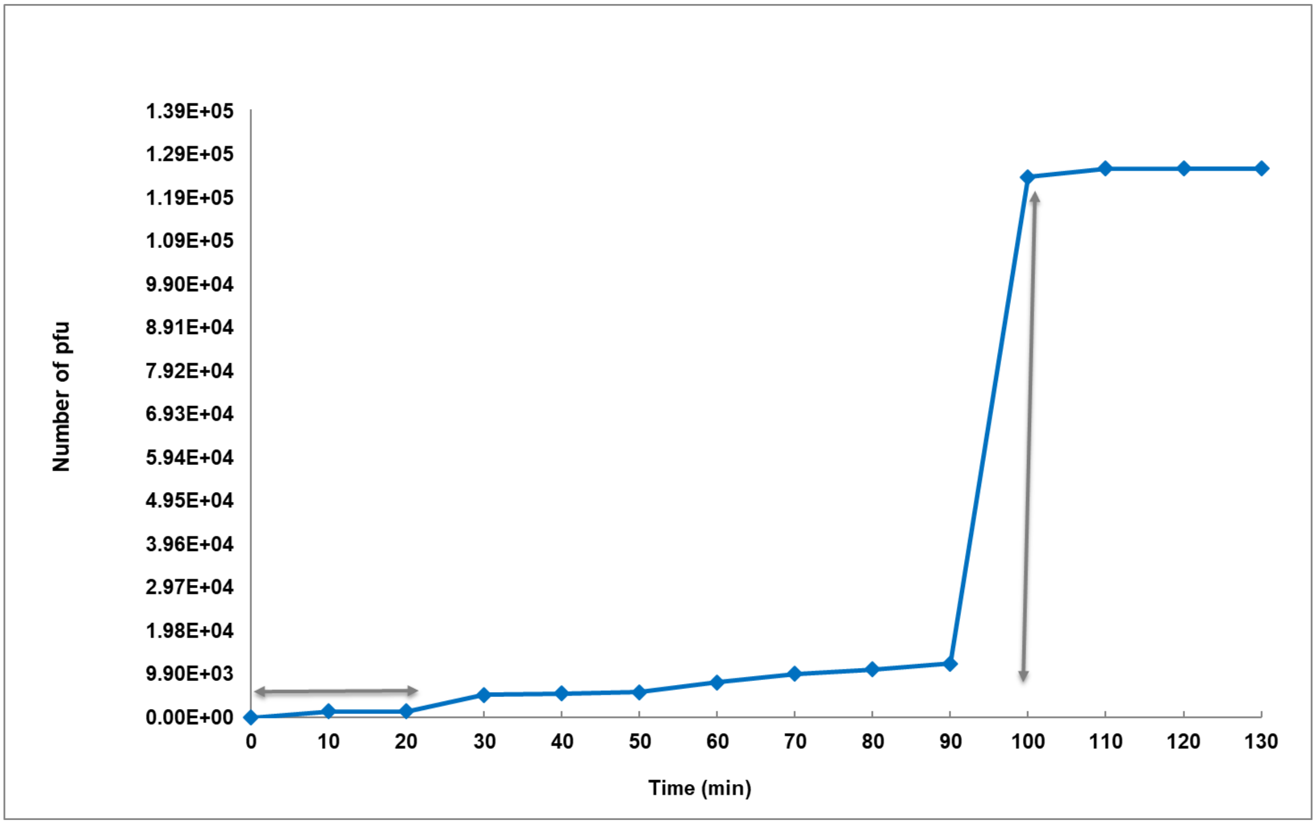

Figure S3. One-step growth of phage M198. Latent period is indicated with a horizontal arrow (20 minutes). Burst size was calculated from timepoint 100 min (indicated with a vertical arrow).
